# Connecting Prognostic Ligand Receptor Signaling Loops in Advanced Ovarian Cancer

**DOI:** 10.1371/journal.pone.0107193

**Published:** 2014-09-22

**Authors:** Kevin H. Eng, Christina Ruggeri

**Affiliations:** Department of Biostatistics and Bioinformatics, Roswell Park Cancer Institute, Buffalo, New York, United States of America; Philipps University, Germany

## Abstract

Understanding cancer cell signal transduction is a promising lead for uncovering therapeutic targets and building treatment-specific markers for epithelial ovarian cancer. To brodaly assay the many known transmembrane receptor systems, previous studies have employed gene expression data measured on high-throughput microarrays. Starting with the knowledge of validated ligand-receptor pairs (LRPs), these studies postulate that correlation of the two genes implies functional autocrine signaling. It is our goal to consider the additional weight of evidence that prognosis (progression-free survival) can bring to prioritize ovarian cancer specific signaling mechanism. We survey three large studies of epithelial ovarian cancers, with gene expression measurements and clinical information, by modeling survival times both categorically (long/short survival) and continuously. We use differential correlation and proportional hazards regression to identify sets of LRPs that are both prognostic and correlated. Of 475 candidate LRPs, 77 show reproducible evidence of correlation; 55 show differential correlation. Survival models identify 16 LRPs with reproduced, significant interactions. Only two pairs show both interactions and correlation (PDGFA
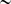
PDGFRA and COL1A1
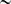
CD44) suggesting that the majority of prognostically useful LRPs act without positive feedback. We further assess the connectivity of receptors using a Gaussian graphical model finding one large graph and a number of smaller disconnected networks. These LRPs can be organized into mutually exclusive signaling clusters suggesting different mechanisms apply to different patients. We conclude that a mix of autocrine and endocrine LRPs influence prognosis in ovarian cancer, there exists a heterogenous mix of signaling themes across patients, and we point to a number of novel applications of existing targeted therapies which may benefit ovarian cancer.

## Background

Signal transduction contains cluses to how abberation in cancer cells may lead to uncontrolled growth and division. It has been hypothesized that evidence of functional signal transduction can be found by examining the mRNA expression-based correlation structure of known ligand-receptor pairs (LRPs) [1]. If correlation is found between a pair, one infers that they form a positive-feedback loop, an autocrine signaling relationship.

A survey in 2006 examined autocrine signaling pairs in epithelial ovarian cancer (EOC) [2] and confirmed their findings by immunohistochemical staining. Importantly, this study continued to develop the idea that differential signaling (a change in autocrine status) might be associated with prognosis.

Statistical differential correlation or differential co-expression (DC) techniques have advanced significantly in recent years allowing for the consideration of multivariate associations beyond treating LRPs one at a time [3]. For example, a Gaussian graphical model (GGM) studies the precision matrix (the inverse of the correlation) to infer signaling and statistical work has developed techniques for proper false discovery control [4]. Recently, it has been proposed to directly estimate the difference in precision matrices to study differential signaling [5].

Combining new correlation techniques with the foreknowledge of candidate signaling pairs derived from protein folding models and confirmatory biochemical experiments [6], we conjectured that a multivariate survey may yield better functional understanding of clinically relevant signaling with strong translational potential. We focus on the Cancer Genome Atlas (TCGA) study of ovarian cancer [7] as a discovery set paired with two large, independent studies for validation [8], [9]. Our plan is an update of the study conducted by Castellano *et al*. (n = 522) in that each of the new datasets totals over 500 patients and comprise a more clinically relevant set – a higher percentage of high-grade EOC and long follow up periods – each conducted as single study instead of several small studies.

We augment the autocrine signaling hypothesis with similar correlation-based models of cancer-relevant signal transduction. Zandi and colleagues reviewed a number of modes of deleterious signaling in the EGFR family of transmembrane receptors [10], relevant to expression data are: the overexpression of the ligand, the overexpression of the receptor, and receptor-receptor crosstalk. In addition to DC analysis, we consider whether the interaction term in a survival regression model can detect an association directly. Finally, we consider whether prognostic LRPs vary over time and heterogenously across patients.

## Methods

### Ligand-Receptor Pair Database

We identified interacting pairs from the Database of Ligand-Receptor Partners (DLRP, http://dip.doe-mbi.ucla.edu/ [1]), matching 162 ligands, 131 receptors and 419 paired interactions on the Affymetrix array. We augmented this set with KEGG pathways [6] hsa04060 (Cytokines/Chemokines) hsa04512 (Cell adhesion molecules) and hsa04514 (ECM interactions). In total, there are 475 pairs (200 ligands, 166 receptors) for consideration after verifying the ligand/receptor functions (Table S1).

### Expression and Clinical Data

Correlation and survival analysis depend on 503 TCGA samples [7] (Table S2) and 500 validation samples collected from two studies by Tothill and colleagues [8] and Yoshihara and colleagues [9]. In each study, measurements are made on tumor tissue excised during surgery prior to chemotherapy. Expression is measured on Affymetrix's HT-HG-U133A and HG-U133 Plus 2.0 arrays (GPL570, GPL571) for the TCGA and Tothill (GSE9899) studies as well as an Agilent platform (GPL56480). Notably the TCGA study uses 3 independent platforms, we opted to analyze just the U133A assay as it was most complete at the time of data acquisition. We follow the standard RMA [11] algorithm and further reduce data to gene level measurements by the brightest-spot rule. Standard gene names are then matched across platforms.

While TCGA samples are selected for advanced serous EOC, Tothill's study contains a representative number of endometriod subtypes; cases with low maligant potential are omitted. Yoshihara's dataset are strictly high-grade, high-stage ovarian cancers. Further, the rate of optimal debulking surgeries is high in the Yoshihara data set (40% vs. 23% p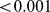, Table 1). Beyond this difference, these patients all undergo debulking surgery and adjuvant platinum-based chemotherapy as standard treatment.

**Table 1:** Clinical differences between discovery and validation studies.

		Studies			
		Discovery	Validation		
		TCGA	Tothill	Yoshihara	
N		503	240	260	
Age	Mean	59.8	60.2	NA	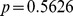
Stage	I, II	24	9	0	
	III, IV	463	215	260	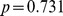 *
Grade	1	4	5	0	
	2,3	473	218	260	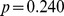 **
Cytoreduction	Optimal	102	53	103	
	Suboptimal	348	140	157	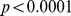 +
Follow Up					
OS	Median	30	28	41.5	
PFS	Median	13.2	14	19	

*excludes Yoshihara data, p-value  = .001597 when including Yoshihara data.

+ definition of optimal/suboptimal not clearly defined in Yoshihara data.

We have focused on progression-free survival (PFS) instead of overall survival as post-recurrence treatments are varied and will confound the genetic signals apparent in pre-treatment tissue. Below, we will stratify patients based on PFS to 18 months, the typical median time to progression for advanced EOC [12]. This is a clinically meaningful endpoint as patients who progress after this time will typically be called “platinum sensitive” and will receive a second regimen of platinum (and taxane) chemotherapy. Standard treatment for patients who recur before this interval is uncertain.

### Models and Hypotheses

We summarize the various LRP models to be tested. Each of these models will have its own specialized analysis. Throughout the paper, we will refer to a specific LRP as Ligand
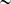
Receptor to emphasize the pairing.

- **Model 1**. Correlation of ligand-receptor pair expression implies autocrine signaling. [1]
- **Model 2**. Correlation in short PFS subset and lack of or inverse correlation in long PFS subset implies prognostic autocrine signaling. [2]
- **Model 3**. Significant statistical interactions crossed by correlated sets should imply functionally relevant signaling.
- **Model 4**. Receptor-Receptor correlation implies receptor cross-talk or heterodimerization. [10]

### Correlation Models

We compute Pearson's correlation between the expression of ligand and receptor genes. Significance tests are based on the statistic 
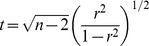
 that follows a t-distribution with n-2 degrees of freedom [13]. Note that correlation is invariant to scaling and centering. To account for the effect of outliers, we performed a sensitivity analysis using Spearman's rank correlation (the effect of a small number of outliers will be attenuated). Our results did not change substantially.

Differential correlation is based on stratifying patients into long PFS intervals (PFS 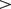
18 months) and short PFS intervals. Patients censored prior to 18 months are excluded from analysis, because these patients have insufficient followup to positively identify them into a long or short response; that is, patients who are known to recur before 18 months are assigned to the short interval group. There are 227 long PFS intervals and 186 short in the TCGA study. For each LRP, we computed correlation in each set separately (Bonferroni 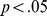) and classified pairs as “both” if long and short PFS sets have significant differences. We imagine that the long PFS subset represents nominal function, so that if a pair is correlated in the long PFS set and not in the short PFS set, we say the effect is a “loss” of function. Conversely, if a set is correlated in the short PFS set and not in the long PFS set we say the effect is a “gain” of deleterious function.

### Survival regression models

For each LRP, we regressed PFS on a set of candidate Cox proportional hazards regression models. In these models, the Ligand and Receptor may act additively or may interact. We selected the model maximizing Akaike's Information Criterion for each pair, classifying them as simply additive or synergistic if the interaction term was significant. We considered both scaled and centered data and expression rescaled to quantiles so that the hazard ratios have a more consistent interpretation. Again, the quantile scale reduces the potential effect of outliers.

### Partial correlation for receptor crosstalk models

We estimated the Gaussian graphical model (GGM) via the GeneNet algorithm with edge posterior probability 0.90 [4], again stratifying patients into long and short PFS subsets. Edges that appear in both sets are classified as functional but not specific to prognosis, edges specific to the short survivors are termed “gain of function” and the edges specific to the long survivors are termed “loss of function.” We harmonized the estimated graphs by combining them with significant LRPs selected through the DC analysis. The graphical layout was done by hand aiming to emphasize clarity and size of the contiguous networks.

### Heterogeneity and predictive models

Our analysis so far assumes homogeneity in time and across patients. We attempt to relax the first by noting that the DC analysis requires us to pick a timepoint to define long/short PFS. We vary this threshold across 1 to 60 months and record the change in DC test statistics. This sensitivity analysis highlights the range of time for which a particular signaling pair is relevant for prognosis.

To test homogeneity in signaling pairs across patients, we select only the LRPs from the DC analysis and run a supervised clustering algorithm to detect subgroups [14]. This algorithm clusters patients based on having similar survival models. We assigned patients to their *maximum a posteriori* class and computed an importance score: the absolute value of the expression level of the interaction multiplied by the cluster specific regression effect for each patient. Large scores reflect the determination that signaling in this LRP has a large influence on a particular patient's prognosis; small values imply little influence.

## Results

### Global correlation and active autocrine signaling

Following Graeber and Eisenberg's hypothesis, we tested for significant correlation between 475 ligand-receptor pairs and found 96 significant pairs in the TCGA discovery set (Bonferroni 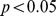). Of these, 77 are reproduced in our validation set with significant correlation and concordant direction of effect (Table S3). Using Spearman's rank correlation to attenuate the effect of outliers, 70 of the 77 are robust to extreme values. Three pairs have significant negative correlation: INHBA
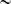
ACVR1B, INHBA
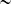
ACVR2B and JAM2
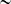
F11R. We note that the magnitude of the selected correlations is remarkably strong (median 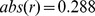 with 13/77, 16% greater than 0.50) which is consistent with previous studies [2]. A list of the full list of significant pairs, their estimated correlation and significance tests in both data sets is included in supplemental material.

For reference, we considered the distribution of sample correlations after permuting the relationship between ligand and receptor. Pairs that were individually significant (p<0.05) had an average correlation of 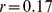 given the true pairings versus and average 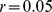 for pairs with significant associations given random associations (t-test p = 4.4e-11). If we randomly select 475 transcript pairs from any measured gene, the average effect drops to 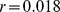 (t-test p = 2.8e-16). This implies that the average direction and magnitude of the observed LRPs are unlikely to be coincidental.

### Ligand-receptor correlation stratified by long/short prognosis

**Figure 1:**
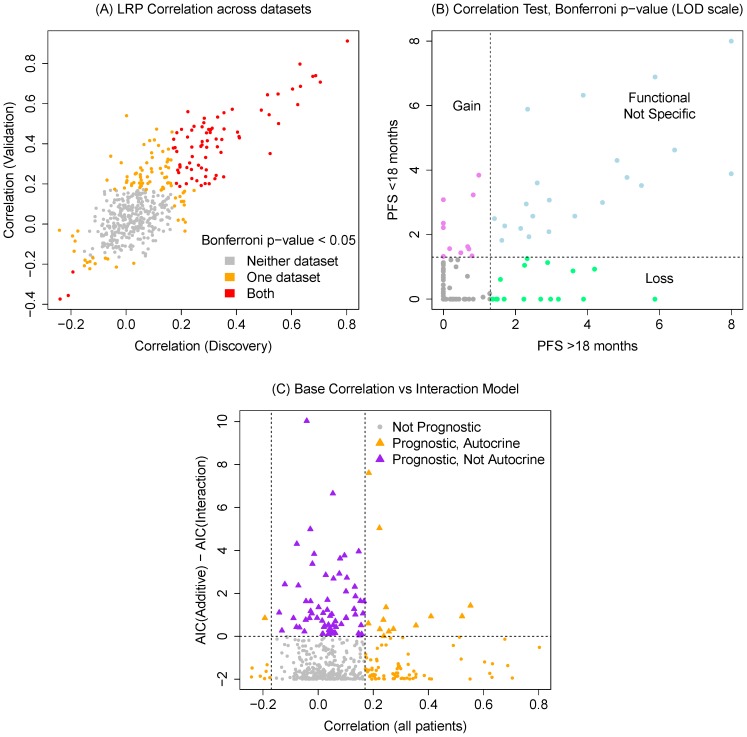
Correlation among ligand-receptor pairs. (A) Correlation in ligand-receptor pairs (LRPs) in the discovery and validation datasets is largely concordant. (B) Stratified by prognosis, there is evidence of differential correlation. (C) The intersection of correlation and survival regression model interactions suggests that some LRPs are prognostic but not necessarily correlated (not autocrine).

Stratifying patients by long and short PFS, we identify 63 pairs with significant correlation in one or both subgroups. Of these 55 are found to have a significant gain or loss in the validation set. We note in Figure 1B, that significant correlations are uniformly positive consistent with a positive feedback loop. A pair can have positive or non-significant (n/s) correlation in the (long; short) PFS strata: we classify them as (positive; n/s), a loss of function; (n/s, positive), a gain of function; (positive, positive) functional but not specific to survival.

Using the discovery cohort, the selected set of pairs comprises 39 receptors and 56 ligands (Table S4). The most frequently represented receptors are FGFR2, FGFR3, and EPHA5, each with at least 5 connections. While most of the remaining ligands are specific to one receptor (34/39, 87%), CCL13, CCL7, CCL8, FGF9, IL15 each form pairs with 3 receptors. With reference to the findings by Castellano and colleagues [2], we find that in at least one dataset, ENFB2
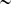
EPHA4 had a gain of function, LIF
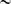
IL6ST had loss of function and IL15
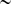
IL2RG had non-specific function. The rest of the pairs had no significant change in correlation in either dataset.

This set is largely concordant with the analysis using all patients together. All pairs labelled with correlation in both sets were previously selected (34 of 63). Three pairs identified by stratified analysis had no significant marginal correlation (JAG2
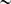
NOTCH3, NGF
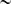
NGFR, and INHBC
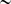
ACVR2B); these pairs would have been missed without stratification. A significant subset of previously identified pairs were not significant in the stratified analysis (36/96, 38%), likely due to a loss of power due to splitting the sample, implying they are likely lower confidence results.

### Ligand-receptor interaction in regression models

No single ligand or single receptor was associated with PFS without considering its paired relationship (Cox regression, Bonferroni-adjusted likelihood ratio test). We subsequently identify significant paired ligands and receptors which supports the hypothesis that signal transduction is associated with survival versus simple overexpression of the ligand or receptor.

Regressing on the quantiles of expression, 29 pairs have selected interaction models (Table S5). Of these, 16 have interaction or additive models in the validation cohort. Four have consistent directions of effects across datasets; three of these are in the ephrin family and one is FGF1-FGFR2. If we consider scaled and centered expression data (Table S6), 9 pairs are again selected and validated: PDGFA
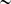
PDGFRA, COL1A1
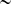
CD44, IFNA14
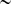
IFNAR2, JAG2
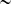
NOTCH2, EFNA3
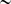
EPHA1, EFNB3
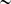
EPHA4, FGF1
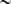
FGFR2, VEGFA
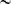
NRP1, and EREG
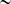
ERBB4.

When cross-tabulated by the correlation analysis, most pairs with significant interaction regression models do not have marginal correlation between the ligand and receptor. Thus, we infer the existence of a class of prognostic signaling pairs that operate without an autocrine feedback loop and we hypothesize that these ligand-receptor pairs act on prognosis in a typical endocrine fashion.

### Graphical model of receptor crosstalk

We considered whether there is evidence of receptor heterodimerization and crosstalk functioning as a mechanism of oncogenic signaling. Continuing to assume that correlation implies signaling cooperativity, we used Gaussian graphical modeling techniques to estimate the graphical edges between receptors. We discovered 29 edges (posterior probability 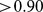) between the 39 receptors with significant differential associations from the stratified analysis. These edges are again classified into Loss/Gain/Non-specific function based on whether they are significant in the long PFS strata, the short PFS strata or both; the majority are losses (22/29, 76%) suggesting a breakdown in signaling coincides with poorer outcomes.

**Figure 2:**
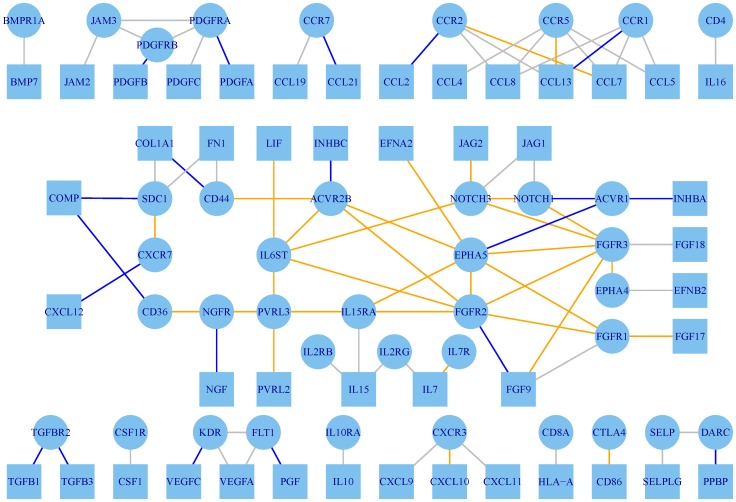
Multivariate correlation graph. Connecting all of the validated LRPs to one another via estimated receptor cross-talk shows a mix of small independent graphs and a large single signaling graph. Grey edges are not specific to prognosis; blue edges are gained in poor prognosis patients.

Figure 2 connects the active ligand-receptor pairs to one another via cooperating receptors. We see that there is one large network of ephrins, the fibroblast growth factor (FGF), and Notch signaling that, as noted, tends to lose its coherence in the short PFS strata. There exists a small CCR chemokine network that is strongly interconnected (13/18 possible edges) indicating both their promiscuity and potential for a multivariate risk phenotype. Other small graphs include a PDGFR family and VEGF/PGF growth factor family. The remaining receptors operate independently.

### Heterogeneity in prognostic signaling

**Figure 3:**
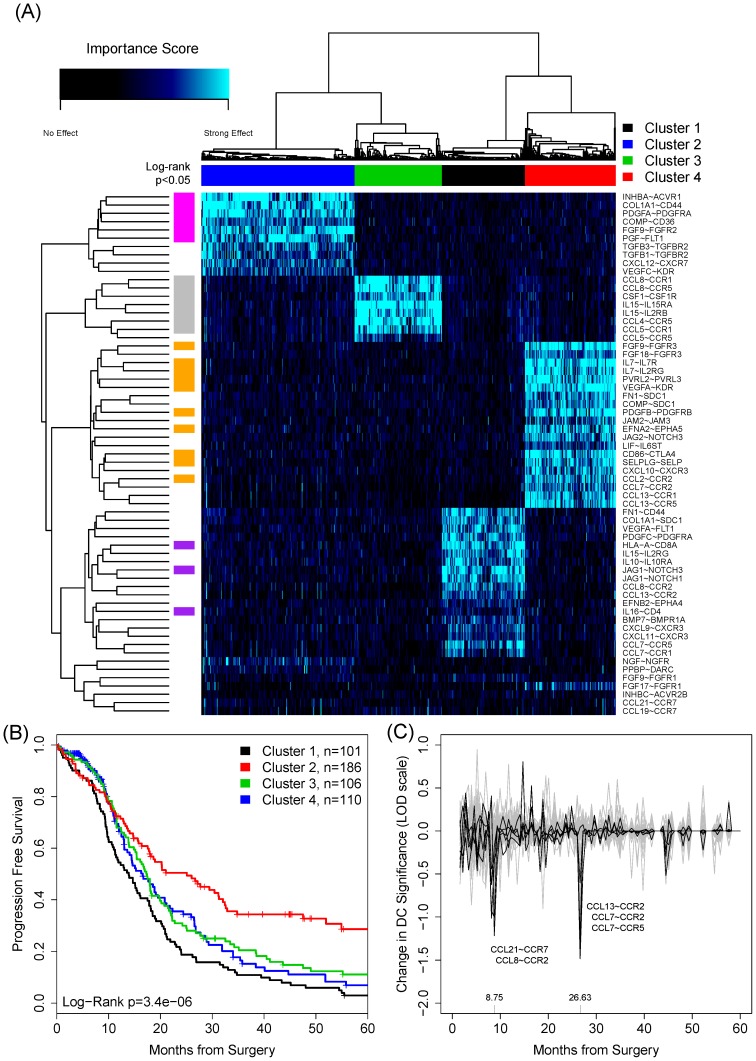
Heterogeneity analysis by patient and in time. (A) Patient heterogeneity implies four prognosis clusterse driven by different LRPs. (B) Each of these clusters has a distinct prognosis. (C) Over time, there are significant changes in the prognostic association and specific LRPs at about 9 and 27 months, close to the second treatment times for platinum resistant and sensitive patients.

To study heterogeneity in signaling across patients, we applied a supervised clustering algorithm to identify prognostic, multivariate sets of LRPs. It identifies four subsets of patients defined by the relationship of signaling pairs and PFS visualized in Figure 3. Patients are arranged by cluster (columns) and LRPs are in rows where LRPs with significant univariate associations with PFS are highlighted.

The first cluster of patients has no relation between signaling and PFS (Cox model 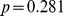) and has the worst prognosis (13.9 vs 18.0 months median, p = 1.8e-05) likely reflecting the fact that this cluster has the highest rate of patients with progressive disease after primary treatment. The second cluster's model has a mild prognostic effect (R^2^ = 0.254, p = 1.0e-06) with 6 significant pairs (PDGFA
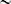
PDGFRA, COL1A
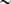
CD44, COMP
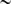
CD36, FGF9
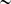
FGFR2, PGF
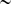
FLT1, INHBA
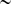
ACVR1). Notably, these interactions are differentially expressed between the first and second clusters implying they may be markers for identifying these patients. The third and fourth clusters are strong models (R^2^>0.600, p<1e-06 for both) with 7 and 10 significant, mutually exclusive LRPs. Patients in the third cluster have significantly shorter PFS (17.3 months versus 25.1, p = 0.00873). Both the longest and shortest PFS groups contain similar signaling pathways; notably the presence of ephrins and FGF differ between them. The models and their clinical associations are summarized in Table 2 with a summary of the drug target relevant signals.

**Table 2:** Clinical associations for signaling clusters.

		Signaling Cluster				
	Plot Color	Black	Blue	Green	Red	
n		101	186	106	110	
Events		100	95	93	73	
PFS Model fit	LRT	0.281	0.001*	0.001*	0.001*	
	R^2^	0.196	0.254	0.620	0.675	
Stage	% III/IV	93%	94%	91%	91%	p = 0.665
Grade	% 3/4	86%	85%	80%	88%	p = 0.420
Clinical	Complete Response	56	106	62	64	
Response	Partial Response	13	17	17	12	
	Stable Disease	2	12	7	5	
	Progressive Disease	14	8	6	8	
Objective Response Rate		68%	66%	75%	69%	p = 0.522
Disease Control Rate		70%	73%	81%	74%	p = 0.292
OS	Median Months	38.3	41.0	44.8	51.9	p = 0.0875
PFS	Median Months	13.9	16.6	17.3	25.1	p<0.001
Notable Effects		VEGF	VEGF	Immune	VEGF	
		PDGF	PDGF		PDGF	
		Ephrins	FGF		FGF	
		Notch	TGF*β*		Notch	

*: p-value smaller than 0.001.

OS: Overall survival.

PFS: Progression-free survival.

LRT: Likelihood-ratio test.

Varying the cutpoint for long/short PFS, we considered whether the DC test statistic was sensitive to a particular time range for each LRP (Figure 3). While all pairs remain significantly DC throughout the range of followup, there are two critical periods where significance drops for several pairs implying they may be more relevant to early followup: at 8.75 months, CCL12
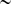
CCR7 and CCL8
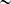
CCR2; and at 26.63 months, CCL13
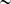
CCR2, CCL7
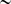
CCR2, and CCL7
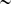
CCR5. Notably, these times seem to correspond to the median time to initiation of secondary therapies for platinum resistant and platinum sensitive cancers suggesting that these cytokines are related to therapy response.

## Discussion

We have extended autocrine signaling loop screening techniques to incorporate survival outcomes more comprehensively. We confirmed the association of ephrin family receptors [2] as well as NOTCH signaling [7] with ovarian cancer prognosis. We find a class of LRPs showing autocrine-type correlation but with no effect on survival; these would have been false-positive associations made in previous studies. Conversely, we find a class of LRPs with survival associations that have no significant correlation which implies their nominal function affects prognosis without any system feedback. In addition, we have shown that mutually exclusive sets of LRPs may function in different programs of both gain and loss of signaling function. The connection between these LRPs might be made via correlated changes between receptors and we have estimated the relevant wiring between them.

Drawn from a solely computational analysis, our conclusions are limited to transcriptome-type studies. Two recent concerns with these studies are run batch effects and the influence of tumor purity. We have used multiple large and independent datasets to reproduce candidate signals to alleviate some of these concerns. While a natural next step is to verify that these transcripts are indeed expressed in these tissues, the finding that they are reproducible using similar probes is promising.

In the context of chemotherapy, assessing the prognostic importance of signal transduction is an appealing avenue for biomarker development relevant for ovarian cancers: nearly 75% of cases eventually fail surgical control with primary platinum/taxane adjuvant chemotherapy [12] and common second-line treatments (gemicitabine [15], topotecan [16]) show differences in immune effect/response.

We have highlighted three particular receptor tyrosine kinases (VEGFR, PDGFR and FGFR) each of which is targeted individually or in combination by one of a number of inhibitors at various stages of approval [17]. Among these compounds are bevacizumab, targeting VEGF signaling [18]; sunitinib, targeting VEGF and PDGF; nintedanib targeting all three; and imatinib targeting just PDGFR. The prevalence of these three signaling interactions leads us to conjecture that different patients may respond to therapies targeting different combinations of receptors which might be inferred from expression data.

We conjecture that the prevalence of immune related LRPs in our validated sets and the per patient heterogeneity suggest the potential for an actionable biomarker to distinguish the utility of chemotherapy or immunotherapy treatment strategies. Further, the discovery of so many immune interactions is likely to be representative of the immunogenic nature of ovarian cancers [19] and of chemotherapy; it is a positive control confirmation for this correlation-based approach.

## Supporting Information

Table S1 **List of ligand-receptor pairs.**
(CSV)

Table S2 **List of TCGA cases used in study.**
(CSV)

Table S3 **Ligand-receptor correlation in TCGA and validation studies.**
(CSV)

Table S4 **Stratified correlation in TCGA and validation studies.**
(CSV)

Table S5 **Regression model selection using quantile scaled data.**
(CSV)

Table S6 **Regression model selection using scaled and centered data.**
(CSV)
